# Integration of ATAC-seq and RNA-seq identifies MX1-mediated AP-1 transcriptional regulation as a therapeutic target for Down syndrome

**DOI:** 10.1186/s40659-023-00474-x

**Published:** 2023-12-09

**Authors:** Zhenglong Guo, Yongchang Zhu, Hai Xiao, Ranran Dai, Wenke Yang, Wei Xue, Xueying Zhang, Bingtao Hao, Shixiu Liao

**Affiliations:** 1 Henan Provincial Key Laboratory of Genetic Diseases and Functional Genomics, Medical Genetic Institute of Henan Province, Henan Provincial People’s Hospital, People’s Hospital of Zhengzhou University, Zhengzhou, China; 2 NHC Key Laboratory of Birth Defects Prevention, Henan Key Laboratory of Population Defects Prevention, Henan Institute of Reproduction Health Science and Technology, Zhengzhou, China; 3 School of Medicine, People’s Hospital of Henan University, Henan University, Zhengzhou, China; 4 Cancer Research Institute, School of Basic Medical Sciences, Southern Medical University, Guangzhou, China

**Keywords:** Down syndrome, Amniocytes, ATAC-seq and RNA-seq, I-IFN, MX1, AP-1

## Abstract

**Background:**

Growing evidence has suggested that Type I Interferon (I-IFN) plays a potential role in the pathogenesis of Down Syndrome (DS). This work investigates the underlying function of MX1, an effector gene of I-IFN, in DS-associated transcriptional regulation and phenotypic modulation.

**Methods:**

We performed assay for transposase-accessible chromatin with high-throughout sequencing (ATAC-seq) to explore the difference of chromatin accessibility between DS derived amniocytes (DSACs) and controls. We then combined the annotated differentially expressed genes (DEGs) and enriched transcriptional factors (TFs) targeting the promoter region from ATAC-seq results with the DEGs in RNA-seq, to identify key genes and pathways involved in alterations of biological processes and pathways in DS.

**Results:**

Binding motif analysis showed a significant increase in chromatin accessibility of genes related to neural cell function, among others, in DSACs, which is primarily regulated by members of the activator protein-1 (AP-1) transcriptional factor family. Further studies indicated that MX Dynamin Like GTPase 1 (MX1), defined as one of the key effector genes of I-IFN, is a critical upstream regulator. Its overexpression induced expression of AP-1 TFs and mediated inflammatory response, thus leading to decreased cellular viability of DS cells. Moreover, treatment with specific AP-1 inhibitor T-5224 improved DS-associated phenotypes in DSACs.

**Conclusions:**

This study demonstrates that MX1-mediated AP-1 activation is partially responsible for cellular dysfunction of DS. T-5224 effectively ameliorated DS-associated phenotypes in DSACs, suggesting it as a potential treatment option for DS patients.

**Supplementary Information:**

The online version contains supplementary material available at 10.1186/s40659-023-00474-x.

## Introduction

Down syndrome (DS), also known as Trisomy 21, is the most frequent chromosomal aneuploidy affecting approximately 1/700 live births [1]. Individuals with DS display a range of clinical presentations including intellectual disability, congenital abnormalities, and in older populations, Alzheimer’s disease (AD) [2]. The clinical phenotypes observed in DS patients are thought to be attributed to the gene dosage effect of an extra chromosome 21 (chr21) [3, 4]. For instance, adults with DS often experience AD symptoms by the age of 40, which is closely associated with the accumulation of amyloid precursor protein (APP), encoded by the *APP* gene in chr21 [5–7]. Individuals with DS are predisposed to multiple diseases, including infections, mucocutaneous abnormalities [8–10], type 1 diabetes mellitus [11], and leukemia [12]. Studies have shown that dosage effect of two genes on chr21, *IFNAR1 and IFNAR2*, encoding two core subunits of the receptor for Type I Interferon (I-IFN), may contribute to mild interferonopathy and drive immune disturbances [13–16]. Additionally, these genes have been implicated in microglial dysfunction [17], and impaired cardiogenesis in DS [18]. However, the underlying molecular mechanisms are not yet fully understood.

Our previous studies have identified that MX Dynamin Like GTPase 1 (MX1), involved in I-IFN activation, may partially contribute to the functional disorders observed in DS derived amniocytes (DSACs) through transcriptome analysis. Interestingly, *MX1* not only belongs to the known I-IFN inducible effector genes [19, 20], but it is also located on chr21, and has been demonstrated shown to be highly expressed in DS [21]. However, the role of *MX1* in DS pathogenesis remains elusive. To understand the interplay between I-IFN and the abnormal phenotypes displayed in DS, we integrated the ATAC-seq and RNA-seq results of DSACs and identified that MX1 may modulate the expression of AP-1 transcription factors (TFs), which affects the transcriptional regulation of genes related to neural dysfunction in DS. T-5224 is a selective inhibitor of c-Fos/AP-1 that specifically inhibits the DNA binding activity of AP-1. It was originally developed as an anti-inflammatory drug to suppress the production of inflammatory cytokines and MMPs [22–26]. Notably, T-5224 treatment significantly improved cellular activity in DSACs.

Our study establishes a link between the dosage effect of MX1 and the AP-1 mediated transcriptional profile in I-IFN driven DS pathogenesis, thereby providing a potential mechanism for the involvement of MX1 in the dosage effect observed in DS. Consequently, AP-1 inhibitors, such as T-5224, may serve as therapeutic candidate for the clinical treatment of DS.

## Materials and methods

### DS derived amniocytes culture and T-5224 treatment

DS and Euploid amniocytes derived from pregnant women in the second trimester with gestational weeks 17–23 were diagnosed through karyotyping from the Prenatal Diagnosis Center of Henan Provincial People’s Hospital (Zhengzhou, China). Amniocytes were cultured in BIO-AMF™-3 Medium (Biological Industries, Beit Haemek, Israel) and maintained at 37 °C in a 5% CO_2_ incubator. To explore the role of inhibiting AP-1 activity on DS, DSACs were treated with 40 µM T-5224 (M9373, Abmole, Houston, USA) for 48 h, and dimethyl sulfoxide (DMSO) treated DSACs were used as NC group.

### ATAC-seq library construction and data analysis

The cultured amniocytes of passage 2 from DS and Normal group were used in this study, in which 3 samples in each group from different fetus were utilized for ATAC-seq and RNA-seq data from 4 different samples from 3 Normal and 4 DS samples were used for integration analysis. All the samples used in this study were male in gender unless noted otherwise. ATAC-seq library was prepared using the TruePrep DNA Library Prep Kit V2 for Illumina (TD501, Vazyme, Nanjing, China). Approximately 50,000 cultured cells were split to obtain nucleus, fragmented with Tn5 transposase followed by PCR amplification. DNA Fragments were purified and sorted using VAHTS DNA Clean Beads (N411, Vazyme, Nanjing, China). Library concentration was quantified through Qubit 3.0, fragment length distribution was examined by DNF915, and libraries were sequenced on Illumina NovaSeq 6000 (Novogene, Tianjin, China). ATAC-seq data analysis was conducted as previously described [27, 28]. Briefly, raw sequence reads were processed by Fastp (v 0.20.0) to remove adapters and low-quality reads. Then the Clean reads were mapped to the human reference genome hg38 using Bowtie2 (v2.3.5.1) with parameters (-sensitiv, -X 2000), followed by removal of PCR duplicated fragments by Picard (v2.22.8). FRiP (fragments ratio in peaks) value was calculated using bedtools (v2.29.2). DeepTools (v3.5.0) was used to generate bigWig file with CPM normalization, which could be visualized in Integrated Genomic Viewer (IGV). The MACS2 (v2.2.4) was used to call peaks, followed by annotated using R package ChIPSeeker (v1.26.0). We analyzed the peak differences between DS and Normal groups using R package DiffBind (v3.10.0). Significant differentially accessible peaks were identified with FDR < 0.05, whose chromosome distribution was analyzed using R package Circlize (v0.4.10) and TF binding motif analysis was conducted through MEME-ChIP online tool (https://meme-suite.org/meme/tools/meme-chip). The promoter was defined as a region within ± 2 kb from the TSS.

### RNA-seq and data analysis

Total RNA of amniocytes from different group was extracted using TRIzol (Invitrogen, Carlsbad, CA, USA) according to the manufacturer’s instructions. After quantification and purification, cDNA library was constructed, and single-end 50-base reads were generated on the MGISEQ2000 platform (BGI-Shenzhen, China). Raw reads were filtered and mapped to human RefSeq using HISAT2/Bowtie2. DESeq2 was used to identify Differentially expressed genes (DEGs) by setting padj (Qvalue) < 0.05 and |log2 (fold change)| ≥ 0.59. Functional enrichment analysis of Gene Ontology (GO), Kyoto Encyclopedia of Genes and Genomes (KEGG) and Gene Set Enrichment Analysis (GSEA) were performed using the online Dr. Tom software (https://biosys.bgi.com), in which Qvalue < 0.05 was used as significance threshold in GO and KEGG analysis, while |NES| > 1, p < 0.05 and FDR < 0.25 were applied to filter enriched pathways in GSEA.

### Establishment of PPI network and TF targets analysis

To build the interaction network of DEGs, the online functional protein association networks analysis platform STRING v.11.5 (https://string-db.org/) was applied [29]. In short, protein names of DEGs were searched in “Multiple Proteins by Names/Identifiers” to establish the protein–protein interactive (PPI) network. The minimum required interaction score was set 0.9 to retain the interacting proteins with high confidence, followed by GO-BP functional enrichment with FDR < 0.05 as the threshold. To predict the Transcription factors (TFs) targeting the expression of DEGs, web-based transcription factor enrichment analysis (TFEA) tool-ChEA3 (https://maayanlab.cloud/chea3/) was utilized [30]. ENCODE ChIP-seq library was used to enrich TFs (FDR < 0.05) associated with submitted genes, followed by tissue specific distribution and GO analysis through GTEx co-expression network Visualizations.

### MX1 overexpression (OE), knockdown (KD) and cell viability detection

Human *MX1* coding sequence (CDS, Gene ID: 4599) was cloned into the lentivirus backbone pcSLenti-EF1-EGFP-F2A-Puro-CMV-MCS-WPRE to construct MX1-OE plasmid (OBIO Biosciences, Shanghai, China). shRNA sequences targeting nucleotide sites of MX1 mRNA were designed, synthesized and inserted into pCLenti-U6-shRNA-CMV-EGFP-F2A-Puro-WPRE to construct MX1-KD plasmid (OBIO Biosciences, Shanghai, China). MX1-OE/KD lentivirus was produced through MX1-OE/KD plasmid transfection into HEK-293 T cells together with the packaging plasmids. Then the amniocytes were infected with MX1-OE/KD lentivirus, followed by 2 µg/mL puromycin (Solarbio, Beijing, China) selection to obtain stable express cells, while the backbone lentivirus or scrambled shRNA lentivirus infected amniocytes were used as control group.

PI (Solarbio, Beijing, China) staining or Cell-Light EdU DNA Cell Proliferation Kit (RiboBio, Guangzhou, China) were applied to detect cell viability according to the manufacturer’s instructions. Hoechst 33342 staining was used to localize the nucleus. The ratio of PI/EdU positive cells to total cells was applied to indicate the cell viability.

### Quantitative realtime PCR (RTqPCR)

Total RNA of amniocytes to be tested was extracted using TRIzol (Invitrogen, Carlsbad, CA, USA) according to the manufacturer’s instructions. Then the quality and concentration of the RNA was determined through Nanodrop (Thermo, Waltham, MA, USA), and cDNA was prepared using RevertAid RT kit (Thermo, Waltham, MA, USA) according to the manufacturer’s instructions. Real-time PCR was performed using an Applied Biosystems StepOnePlus qPCR system with Taq Pro Universal SYBR qPCR Master Mix (Q712, Vazyme, Nanjing, China). The utilized primers were synthesized from Tsingke Biological Technology (Beijing, China), which were listed in Additional file 2: Table S1. 2^−ΔΔCt^ method was used for the quantitation of mRNA expression and housekeeping gene actin beta (ACTB) was used for internal control for normalization.

### EGR1 binding motif analysis within the promoter region of AP-1 TFs by JASPAR

The Cistrome Data Browser (DB) is a resource of published human and mouse ChIP-seq data (http://cistrome.org/db) [31]. In our study, EGR1 ChIP-seq data (CistromeDB: 46169) from human Embryonic Stem Cell (ESC) line-H1 was visualizd in WashU Brower [32]. Then we downloaded the sequence of candidate binding peaks within the promoter region of AP-1 genes including *FOSB*, *JUNB* and *JUND*, followed by prediction through Scan Tool in JASPAR, which provided the information of relative score and location of potential EGR1 binding motifs (https://jaspar.genereg.net/).

## Results

### DS-derived amniocytes exhibit a global increase in chromatin accessibility

**Fig. 1:**
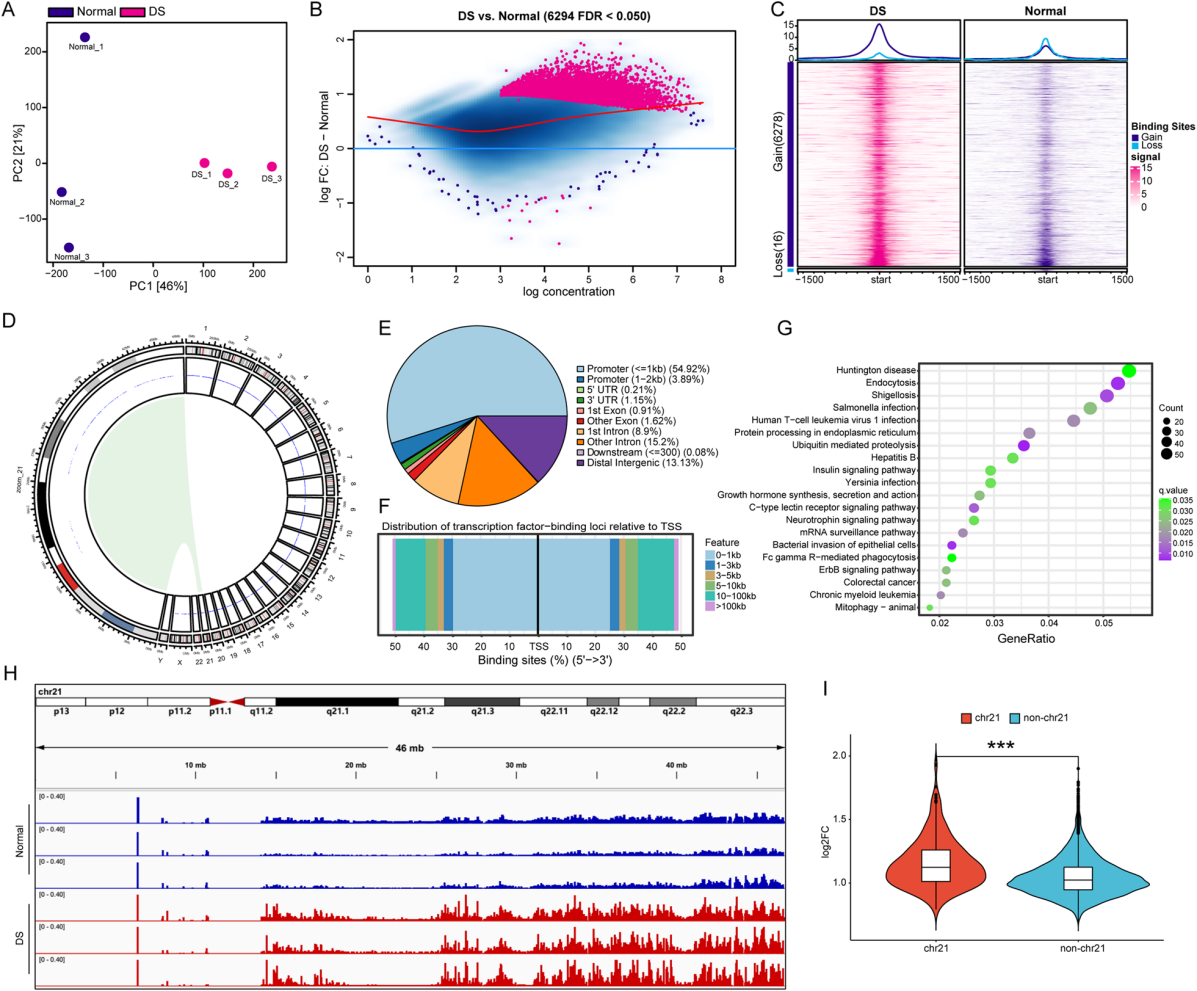
ATAC-seq results in DS derived amniocytes. **A** Principal Components Analysis (PCA) plot. **B** MA plot of DS-Normal contrast. Sites identified as significantly differentially bound shown in red. Red dots above and below the blue horizontal line means gain and loss binding affinity, respectively. **C** Enrichment of ATAC-seq signals around the 1.5 kb upper and lower reaches of transcriptional start site (TSS) with sample groups merged. **D** Chromosome distribution of differential peaks. **E** Location distribution of upregulated peaks on the genome. **F** Distribution ratio of upregulated peaks at the distance from TSS. **G** KEGG enrichment of upregulated peaks. **H** IGV shows peaks on chromosome 21 (chr21) in DS and Normal group. **I** Violin plot of log2FC distribution of differential peaks on chr21 and non-chr21 (***p < 0.001, two-tailed Student’s t-test)

ATAC-seq was performed to detect alteration in accessible chromatin in DS-derived amniocytes (DSACs) compared to controls. Following the processing of raw sequence reads, normalized read counts were utilized for further analysis. To assess the quality of ATAC-seq, Principal Components Analysis (PCA) was conducted, which resulted in the clustering of samples into two groups, indicating differential chromatin accessibility landscape in DSACs (Fig. 1A). Subsequently, differential chromatin accessibility analysis identified a total of 6294 peaks with an FDR < 0.05 (Fig. 1B). Interestingly, 6278 peaks were gained while only 16 peaks were lost in DSACs (Fig. 1C). To gain further insight into the function of these peaks, particularly the gained peaks, we mapped them to human genome (hg38). As depicted in Fig. 1D, the alterations in accessible chromatin were not limited to chromosome 21 (chr21). Specifically, 58.81% of the annotated 6278 gained peaks were specially located in the promoter regions, with a distribution tendency of 0–1 kb away from the transcription start sites (TSSs) (Fig. 1E, F). These findings reveal a significant increase in chromatin accessibility in DSACs, which has recently been observed in human induced pluripotent stem cells (iPSCs) and iPSC-derived neural progenitor cells (NPCs) in DS [33].

Then 3436 genes with increased accessibility of promoter regions (Additional file 3: Table S2) were subjected to KEGG pathway enrichment analysis. Our data showed that pathways related to brain development and function, such as Huntington disease, Endocytosis, Axon guidance and Neurotrophin signaling pathway, were enriched, which was in line with Neurodegenerative changes observed in DS patients (Fig. 1G). Meanwhile, Bacterial and viral infection related pathways were similarly enriched including Shigellosis, Salmonella infection, Human T-cell leukemia virus 1 infection, Hepatitis B, Yersinia infection, Fc gamma R-mediated phagocytosis, C-type lectin receptor signaling pathway and Acute myeloid leukemia, which may reflect dysfunction of the immune system reported in DS. Besides, cell adhesion related pathways were also enriched including Adherens junction and Regulation of actin cytoskeleton (Fig. 1G, Additional file 4: Table S3). These results indicate that genes with increased chromatin accessibility may participate in DS-associated cellular dysfunction.

Due to the presence of an extra chromosome 21 in individuals with DS, we expected to observe obvious increase in the ATAC-seq signals specifically on chromosome 21 in DSACs. As expected, this led to the observation of increased peaks (Fig. 4H). Out of the 6,278 gained peaks, a total of 277 peaks were located on chr21. Quantitative analysis demonstrated a significantly increase in chromatin accessibility of the peaks on chr21 when compared to the peaks on non-chr21 (Fig. 4I).

### Enrichment of AP-1 transcription factor binding motifs was in the gained chromatin accessible regions

**Fig. 2:**
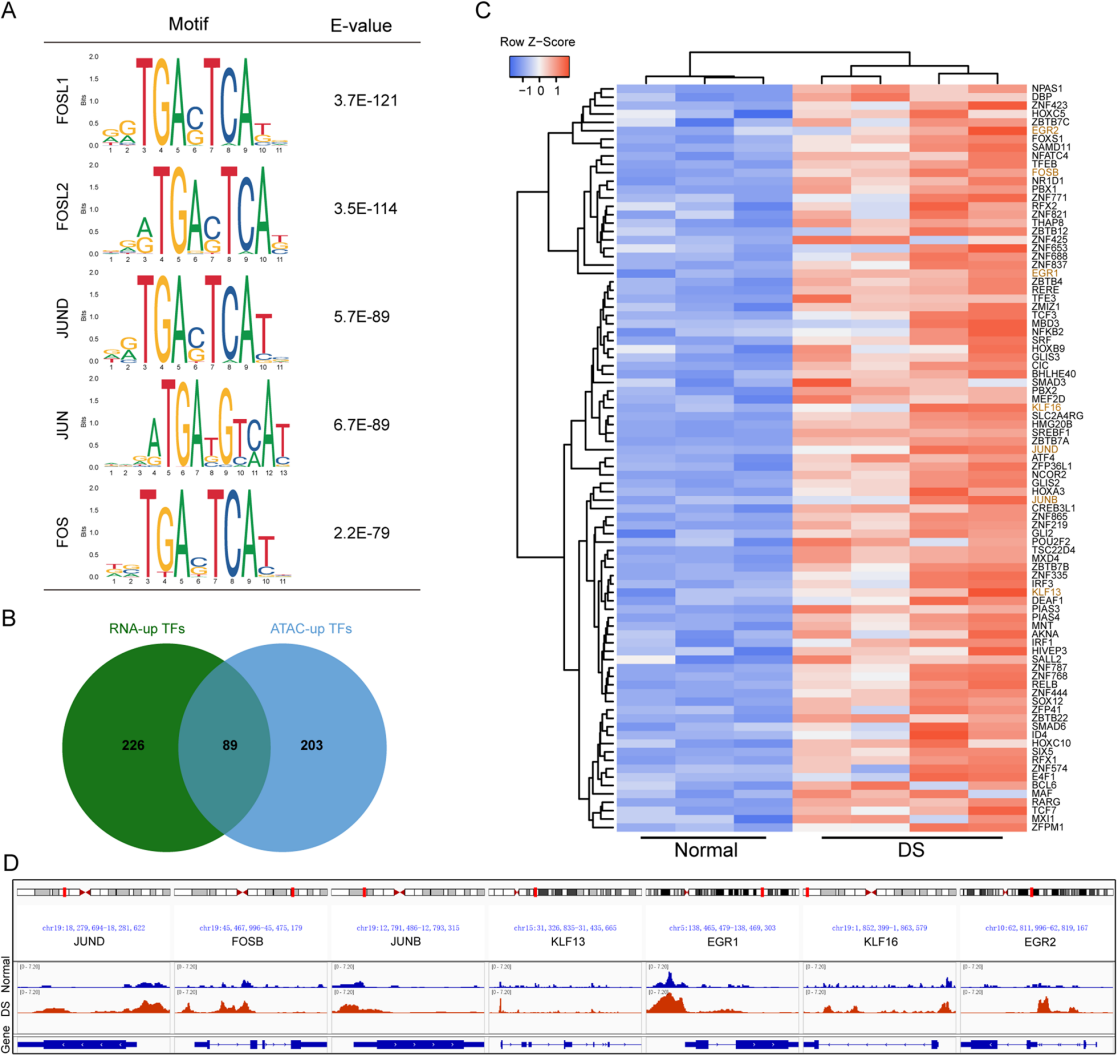
Transcription factor binding motifs enrichment in differential open chromatin regions. **A** The top 5 predicted binding motifs in the comparison between Normal and DS group. **B** Venn plot of the expression of enriched transcriptional factors in RNA-seq and ATAC-seq. **C** Heatmap of the expression of significantly upregulated 89 transcriptional factors in RNA-seq and ATAC-seq. **D** ATAC-seq tracks in the upstream promoter region of 7 key transcription factors in Normal and DS group

To identify candidate transcription factors (TFs) binding to the gained peak regions and potentially regulating gene expression, motif analysis was performed. The top five enriched TF binding motifs exclusively belonged to the AP-1 transcription factor family (Fig. 2A, Additional file 5: Table S4). Through a combined analysis of upregulated TFs in RNA-seq and ATAC-seq, 89 TFs were obtained and displayed in the heatmap of mRNA expression (Fig. 2B, C). To confirm the key TFs, we combined these TFs with candidate motifs and finally 7 TFs were selected including AP-1 (JUND, FOSB, JUNB), KLF13, KLF16, EGR1 and EGR2, which were visualizd in ATAC-seq tracks (Fig. 2D).

In order to account for the impact of gained chromatin accessibility on chromosome 21 in the binding motif analysis, we excluded the peaks specific to chromosome 21 and reanalyzed the data. Remarkably, AP-1 transcription factors (TFs) remained the top enriched TFs even after this exclusion. These findings suggest that AP-1 TFs may play a potential role in abnormal transcriptional regulation in DSACs.

### Integration of ATAC-seq and RNA-seq results

**Fig. 3:**
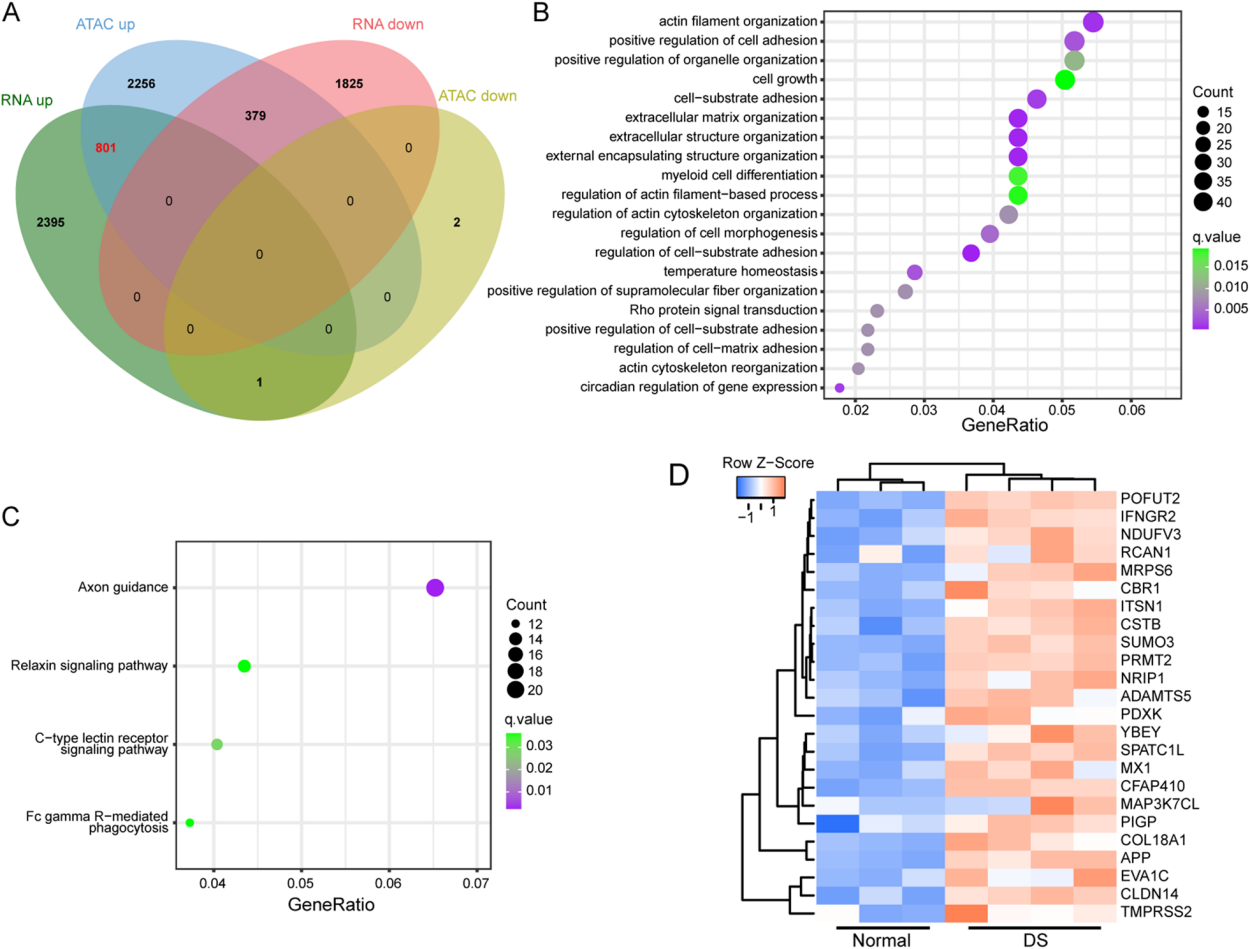
Integration analysis of DEGs in ATAC-seq and RNA-seq. **A** Venn diagram of DEGs in ATAC-seq and RNA-seq. **B** GO-BP analysis of upregulated 801 DEGs. **C** KEGG enrichment of upregulated 801 DEGs. **D** Heatmap of the expression of significantly upregulated genes on chr21

Previously we analyzed the difference of mRNA expression profile between DSACs and Normal Acs through RNA-seq. To study the relationship between chromatin accessibility alteration and changes in gene expression, we performed integrative analysis of RNA-Seq and ATAC-Seq. In general, the RNA-seq DEGs contained 3197 upregulated genes and 2204 downregulated genes (Additional file 6: Table S5), while the ATAC-seq DEGs contained 3436 upregulated genes and 3 downregulated genes. Moreover, integration of ATAC-seq and RNA-seq data defined that 801 genes were simultaneously upregulated in chromatin accessibility and gene expression (Fig. 3A, Additional file 6: Table S5). GO-BP analysis of these 801 genes suggested that multiple terms were changed in DSACs, especially for cell adhesion related pathways, such as Actin filament organization, Cell-substrate adhesion, Extracellular matrix organization, Extracellular structure organization and KEGG analysis result showed that Axon guidance was the most significant enriched pathway, which was closely related to nerve cell function (Fig. 3B, C, Additional file 7: Table S6).

Dosage effect of genes on chr21 may explain DS phenotypes. Actually, 24 out of 801 genes were located on chr21, in which some genes had been verified contributing to neurodevelopmental dysfunction in DS, such as *APP*, *ITSN1*, *NRIP1*, etc. (Fig. 3D). Our previous studies identified that decreased expression of m6A methyltransferase like 3 (METTL3) may lead to global reduction of m6A modification, which result in abnormally increased expression of *NRIP1* in fetal brain cortex tissue of DS [34]. To summarize, our results indicate that increased expression of genes including those on chr21 may play an important role in pathogenesis of DS.

### MX1 function in AP-1 TFs involved regulatory networks

**Fig. 4:**
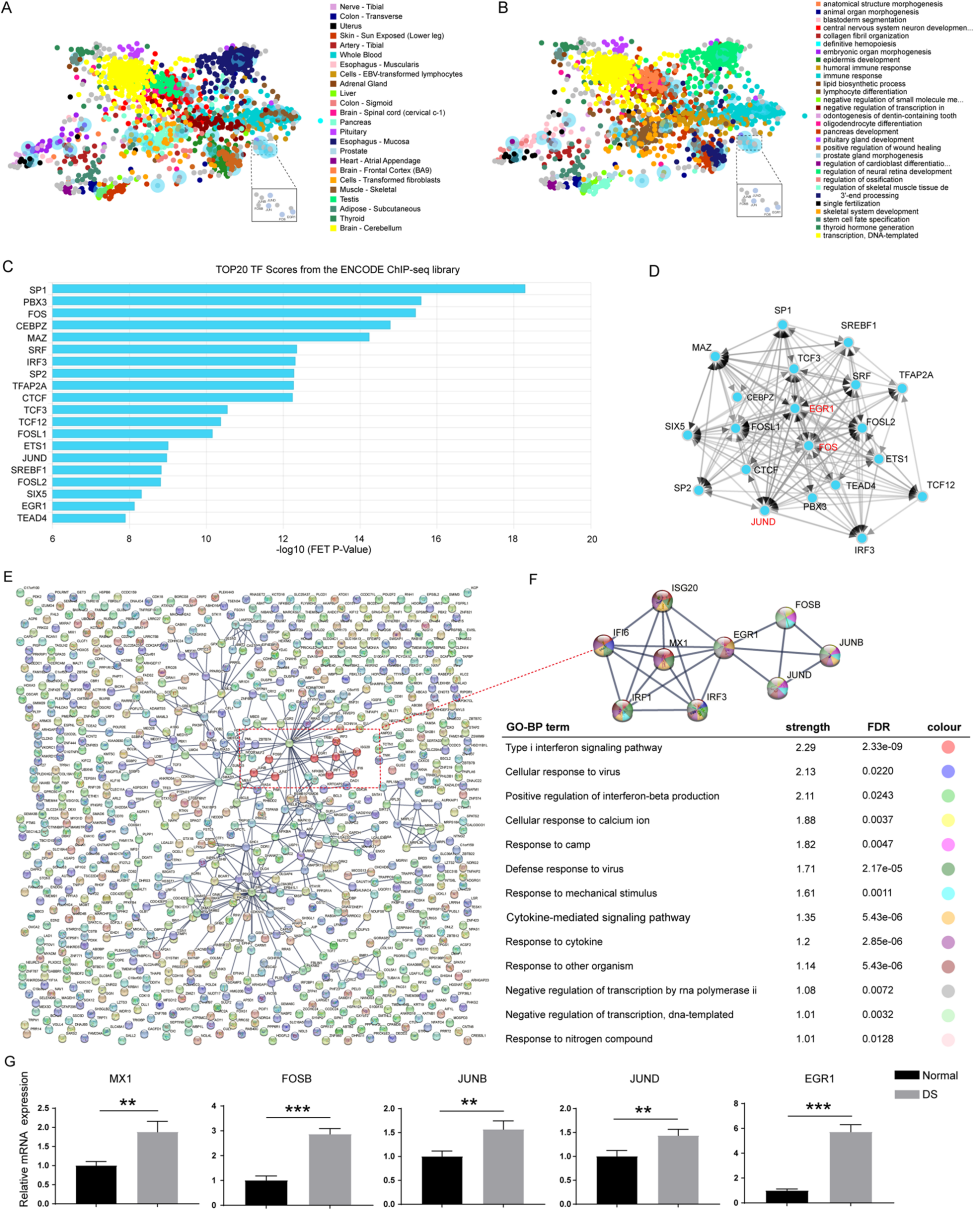
Transcriptional regulation Networks analysis in DS. **A** Tissue distribution of the transcription factors targets for DEGs. **B** GO-BP analysis of the transcription factors targets for DEGs. **C** The top 20 transcription factors targets for DEGs from ENCODE ChIP-seq library. **D** The network of top 20 transcription factors targets for DEGs. **E** STRING protein network analysis of upregulated DEGs. **F** GO-BP analysis of the core regulatory network involving MX1 on chr21 and AP-1 TFs. **G** Quantitative measurement of mRNA expression levels of key genes including MX1 and AP-1 in Normal and DS group (n = 4, **p < 0.01, ***p < 0.001, two-tailed Student’s t-test)

To gain further insights into the TFs responsible for regulating the 801 DEGs, we utilized the ChEA3 tool, an online transcription factor enrichment analysis (TFEA) tool. We analyzed the distribution and biological functions of the enriched TFs (Additional file 8: Table S7). Figure 4A shows that the top 50 enriched TFs from the ENCODE ChIP-seq library were found to be associated with various tissues, such as skin, whole blood, lymphocytes, muscle, and brain. GO-BP enrichment analysis revealed that these TFs were involved in a range of functions, including central nervous system neuron development, regulation of skeletal muscle tissue development, positive regulation of wound healing, thyroid hormone generation, and embryonic organ morphogenesis (Fig. 4B). Notably, consistent with the ATAC-seq TF binding motif enrichment results, AP-1 TFs were also identified. The TF Co-regulatory Networks analysis highlighted the central regulatory role of AP-1 TFs, including FOS, JUN, and JUND (Fig. 4C, D).

In order to identify potential upstream genes involved in regulating the expression of AP-1 transcription factors, we utilized the STRING PPI (Protein–Protein Interaction) online tool. This allowed us to establish an association network among the proteins encoded by the defined 801 genes. After applying conditional qualification, we retained the AP-1 TFs-associated network, primarily composed of JUND, JUNB, FOSB, MX1, and EGR1 (Fig. 4E). Furthermore, MX1, an interferon target gene located on chromosome 21, emerged as a key candidate involved in the regulation of EGR1 and AP-1 TFs expression, as indicated by GO-BP analysis (Fig. 4F). Additionally, RT-qPCR data confirmed the expression of the selected genes in DSACs compared to control cells, highlighting the significant role of MX1 as a mediator of AP-1 expression and transcriptional regulation in DS (Fig. 4G).

### MX1 overexpression induced AP-1 activation and reduces viability of normal ACs

**Fig. 5:**
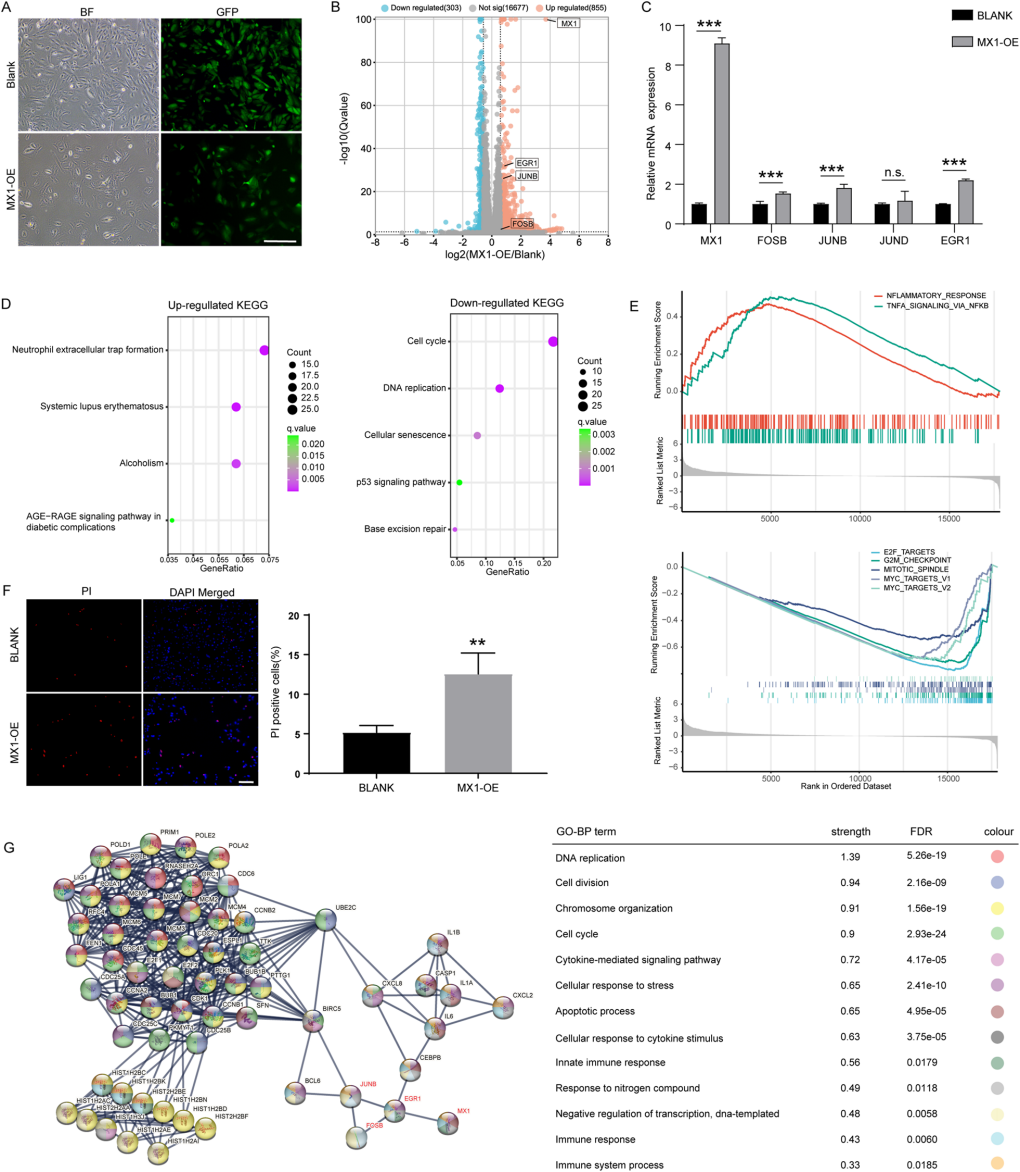
Functional study of MX1 over-expression. **A** Bright field and fluorescence images of normal ACs transfected with MX1-OE and Blank lentivirus (scale bar = 100 μm). **B** Volcano plot of all expressed genes in MX1-OE versus Blank. DEGs were filtered through the Fold change ≥ 1.5 and Qvalue ≤ 0.05. **C** Quantitative measurement of mRNA expression levels of key genes including MX1 and AP-1 in MX1-OE and Blank group (n = 4, ***p < 0.001, two-tailed Student’s t-test). **D** KEGG enrichment of up- and down-regulated DEGs. **E** Gene set enrichment analysis (GSEA) revealed altered pathways related to MX1-OE. **F** PI staining and quantitative results showed proportion of apoptotic cells in MX1-OE and Blank group (scale bar = 100 μm) (n = 3, **p < 0.01, two-tailed Student’s t-test). **G** STRING protein network analysis and GO-BP enrichment of DEGs

To determine the function of MX1 in DSACs, an MX1 overexpression (OE) study was conducted in normal ACs. After lentivirus infection, the cells exhibited relatively slow proliferation (Fig. 5A). Meanwhile, the RNA-seq analysis revealed 1158 DEGs (Fold Change ≥ 1.5 and Qvalue ≤ 0.05), comprising 855 upregulated genes and 303 downregulated genes, in MX1-OE versus control group (Fig. 5B, Additional file 9: Table S8). Interestingly, MX1, EGR1, JUNB and FOSB were all found to be significantly upregulated among the DEGs, with the exception of JUND (Fig. 5C). It is worth noting that although the fold change of FOSB was slightly lower than 1.5 (~ 1.48), this finding was consistent with results obtained from RT-qPCR assay. To gain further insights into the phenotype alterations mediated by MX1 overexpression related to DS, we conducted functional enrichment analysis of the DEGs. The results from KEGG and GSEA analyses revealed that cell cycle-related pathways were suppressed, while inflammation-related pathways were activated (Fig. 5D, E).

During the culture of DS-derived cells, including DSACs, decreased cellular viability is a prominent phenotype. To investigate this further, we assessed the level of apoptosis in the MX1-OE and control groups. Figure 5F shows that MX1-OE cells exhibited a higher level of apoptosis compared to the control group, as observed through PI staining. This suggests that MX1 overexpression diminishes cell viability in normal ACs.

To gain more insights into the relationship between MX1 overexpression and phenotype changes, such as inflammation activation and decreased cell activity, we utilized the STRING PPI network and performed GO-BP analysis. As shown in Fig. 5G, the expression pattern of MX1 and AP-1 TFs was established, highlighting their association with phenotype changes. Moreover, the TF targets analysis of the DEGs revealed the enrichment of AP-1, further supporting its role in the regulatory network (Additional file 1: Fig. S1A, B).

In conclusion, our findings propose a novel mechanism by which MX1 induces DS-like phenotypes through the activation of AP-1 expression. This provides valuable insights into the underlying molecular mechanisms of DS pathology.

### AP-1 specific inhibition attenuates DS-associated phenotype

**Fig. 6:**
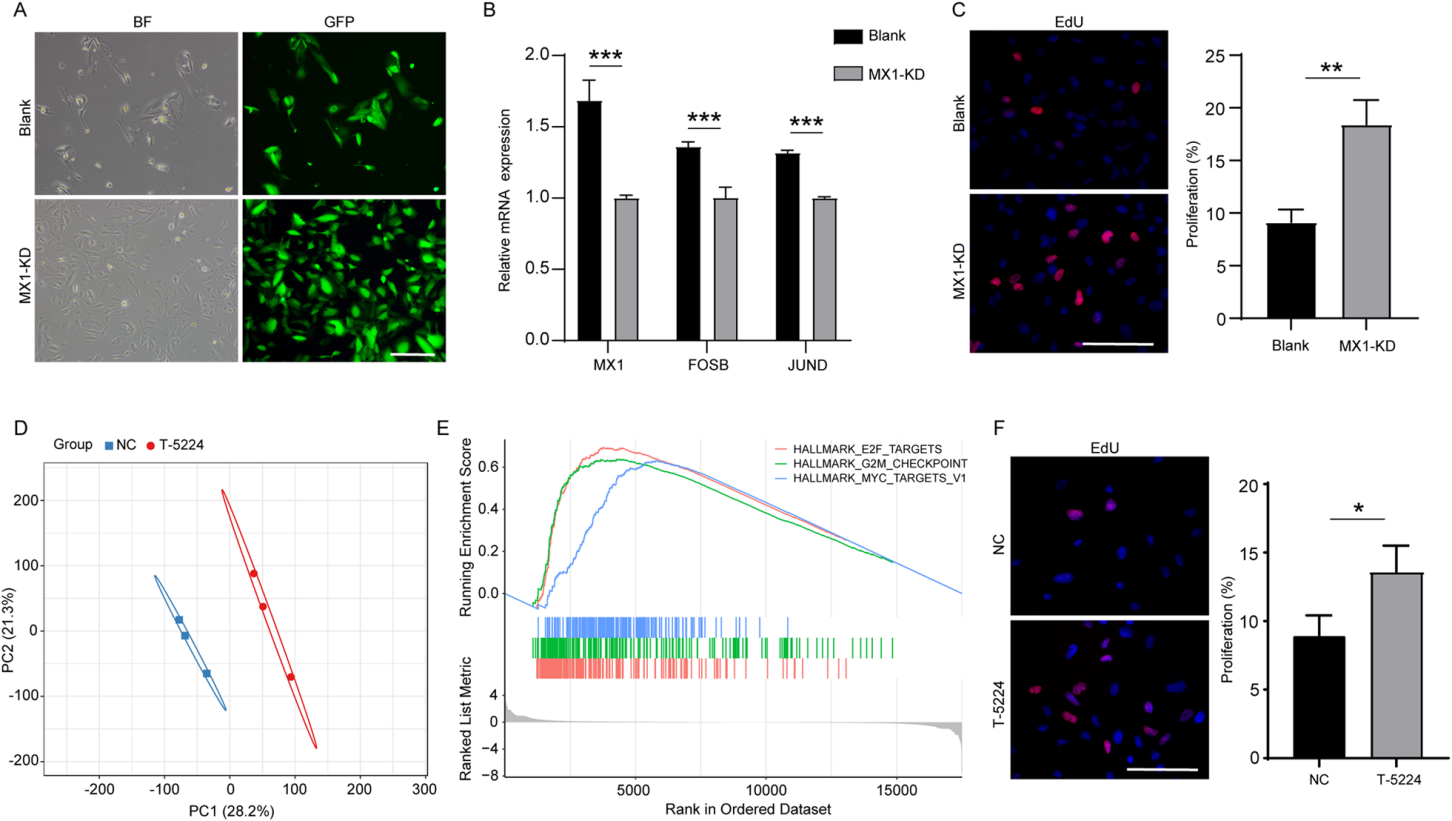
MX1-KD and AP-1 inhibition on DSACs. **A** Bright field and fluorescence images of DSACs transfected with MX1-KD and Blank lentivirus (scale bar = 100 μm). **B** Quantitative measurement of mRNA expression levels of key genes including MX1 and AP-1 in MX1-KD and Blank group (n = 4, ***p < 0.001, two-tailed Student’s t-test). **C** EdU staining and quantitative results in MX1-KD and Blank group (n = 3, **p < 0.01, two-tailed Student’s t-test). **D** Principal Components Analysis (PCA) plot. **E** Gene set enrichment analysis (GSEA) revealed altered pathways in T-5224 treatment versus non-treated DSACs. **F** EdU staining and quantitative results in T-5224 treatment versus non-treated DSACs (n = 3, *p < 0.05, two-tailed Student’s t-test)

Since the upregulation of MX1 may play an important role in AP-1 activation, thus leading to cellular dysfunction, especially lower cell activity in DS, MX1 knockdown (KD) may ameliorate DS-associated phenotypes. After infection of MX1-KD lentivirus, DSACs showed a prominent increase in cell proliferation ability, which could be confirmed through EdU staining results (Fig. 6A, C). Furthermore, downregulation of MX1 expression could significantly suppress AP-1 expression in DSACs, indicating core function of MX1 mediated AP-1 activation in the pathogenesis of DS (Fig. 6B). To assess the potential impact of inhibiting AP-1 activity on DS-associated phenotypes, we treated DSACs with 40 µM T-5224, a pharmaceutical inhibitor specifically designed to target AP-1. As expected, PCA results obtained from RNA-seq showed that T-5224 treatment resulted in changes of gene expression profile (Fig. 6D). Furthermore, GSEA enrichment and EdU results demonstrated the improvement of cell proliferation in T-5224 treated DSACs, indicating that T-5224 had a profound effect on DS treatment (Fig. 6E, F). These findings suggest that specific inhibition of AP-1 through T-5224 represents a promising approach for mitigating DS-associated phenotypes.

## Discussion

Down syndrome (DS), firstly described by John Langdon Down in 1866, is the most common chromosomal abnormality to data [35, 36]. It is characterized by varying degrees of intellectual disability and a range of phenotypic abnormalities affecting multiple organ systems, including the cardiovascular system, skeletal muscular system and respiratory system [2]. The presence of an extra copy of chromosome 21 (chr21), encompassing approximately 200 protein-encoding genes, has long been recognized as the primary causative factor in DS. Among these genes, DYRK1A, DSCR1, HMGN1, APP, and others, have been extensively studied in cellular and animal models, highlighting their contributions to the clinical manifestations of DS [37–40]. Consequently, various therapeutic approaches have been proposed based on our growing understanding of the underlying pathogenesis. These include gene therapy [41], hormonal supplementation [42], senolytic drugs [33], and medications for treating mental illness and Alzheimer’s disease [43]. However, the translation of these promising findings into viable clinical treatments for DS has been frustratingly slow, leaving a significant gap in effective therapeutic options for individuals with DS.

ATAC-seq technology has been developed to investigate the epigenomic profiling of open chromatin regions, thereby providing information about transcription factors regulating gene expression [44]. The integration of ATAC-seq and RNA-seq data allow for the establishment of correlations between alterations in chromatin-accessibility and differential expressed genes, facilitating the construction of the gene expression regulation mechanism involving TF binding. In this study, we utilized amniocytes, which originate from the fetal stage. Interestingly, the ATAC-seq results revealed a global increase in chromatin accessibility, particularly for promoter regions. ATAC-seq results of DS-derived iPSCs and NPCs also showed global chromatin-accessibility changes in response to the additional chr21, despite that the more accessible regions are not predominantly localized within the promoters [33]. Chromatin remodeling is associated with DNA methylation, histone modification and RNA modification [45]. In a previous study, we explored the alteration of RNA m6A modification and its potential role in dysregulated gene expression and DS pathology [34]. Moreover, we established the transcriptome profile of DSACs using RNA-seq, which revealed significant upregulation of histone modification related genes, including *H2BC4*, *H2BC5*, *H2AJ*, *H2AC6*, *H2BC12*, *H2BC2*, *H3C4*, *HDAC7* and *HDAC10*. Meanwhile, Mediator subnit, MED23, was included in the upregulated DEGs as well, which interacts with activators to stimulate transcription initiation [46]. Taken together, these findings may explain global chromatin-accessibility increasements observed in DSACs. The differential chromatin accessibility was observed throughout the genome, with only approximately 4.4% of the increased peaks located on chr21. However, the log2 fold change distribution of the chr21 peaks was significantly higher than that of non-chr21 peaks (Fig. 1). Furthermore, binding motif analysis of the gained ATAC-seq peaks identified AP-1 TFs as potential key regulators in regulating downstream gene expression (Fig. 2). As expect, the upregulated DEGs in both ATAC-seq and RNA-seq dataset comprised 801 genes, including many genes associated with inflammation and brain function related pathways. Within this group, MX1, as part of the Type I Interferon (I-IFN) pathway, was found to potentially monitor the expression of AP-1 TFs, suggesting a role of I-IFN in DS-associated dysfunction (Figs. 3 and 4).

Notably, individuals with DS have been reported to exhibit an overactive I-IFN response, leading to cytokinopathy, hyperactivated T cells and B cells and an increased risk of severe infectious diseases and autoimmunity [47]. Consistently, MX1 overexpression resulted in the significant upregulation of AP-1 TFs, inflammation response, and DS-associated cellular dysfunction (Fig. 5). It is worth mentioning that MX1, located on chr21, is a downstream effector-gene of I-IFN, and associated with Alopecia areata (AA) in DS [48]. EGR1, a zinc finger DNA-binding protein, has also been implicated in viral infection and immune response, and has shown link to I-IFN in recent studies [49, 50]. Additionally, EGR1 binding motifs were enriched within the promoter region of AP-1 TFs, suggesting that MX1 may monitor AP-1 expression through EGR1 (Additional file 1: Fig. S1C–E). Taken together, the dosage effect of MX1 may activate AP-1-mediated transcriptional profiling, resulting in alterations in biological processes and pathways. These findings provide new evidence for the I-IFN-driven pathogenesis of DS.

AP-1, which is a collective name for dimeric transcription factors, predominantly consists of members from the JUN and FOS families. These TFs play crucial roles in modulating various biological processes associated with cell proliferation, differentiation and transformation [51]. Additionally, AP-1 directly regulates the expression of inflammatory cytokines and matrix-degrading matrix metalloproteinases (MMPs), which are involved in the pathogenesis of inflammation diseases and have emerged as new therapeutic targets, particularly in conditions like rheumatoid arthritis [52–55]. Notably, it is intriguing to observe that multiple genes of MMPs and inflammation cytokines were prominently upregulated in DSACs including MMP7, MMP11, MMP13, MMP15, MMP17, IL-1β, TNF-a, and others. In our study, we utilized the AP-1 inhibitor T-5224, which yielded the expected improvement in cell activity, thus highlighting the therapeutic potential of AP-1 inhibition in the treatment of DS. However, it is important to note that DSACs may not fully recapitulate the in vivo disease status. Therefore, further investigations utilizing DS-derived induced pluripotent stem cells (iPSCs) and animal models, in combination with additional functional tests, may provide more definitive conclusions regarding the role of AP-1 and potential therapeutic interventions for DS.

### Supplementary Information

**Additional file 1: Figure S1.** TF targets analysis of DEGs in MX1-OE and EGR1 binding motif in AP-1 promoter region.

**Additional file 2: Table S1.** Primer sequences used for RT-qPCR.

**Additional file 3: Table S2.** Genes with increased accessibility of promoter regions.

**Additional file 4: Table S3.** KEGG pathway enrichment analysis of upregulated genes in ATAC-seq.

**Additional file 5: Table S4.** Binding motif analysis of the gained peak regions.

**Additional file 6: Table S5.** Differentially expressed genes identified from RNA-seq.

**Additional file 7: Table S6.** GO-BP enrichment of the defined 801 genes from integration analysis of ATAC-seq and RNA-seq.

**Additional file 8: Table S7.**Candidate transcription factors targeting the expression of DEGs.

**Additional file 9: Table S8.** Differentially expressed genes identified from MX1-OE studies.

## Data Availability

The clean or raw data of RNA-seq and ATAC-seq used in this study have been deposited into CNGB Sequence Archive (CNSA) [56] of China National GeneBank DataBase (CNGBdb) [57] with accession number CNP0003748 and CNP0004570, and Gene Expression Omnibus database with accession number: GSE237539, and GSE237545.
