# Supplementary material for: Integration of ATAC-seq and RNA-seq identifies MX1-mediated AP-1 transcriptional regulation as a therapeutic target for Down syndrome
Source: Biol Res. 2023 Dec 9;56:67. doi: 10.1186/s40659-023-00474-x (PMC10709892; doi:10.1186/s40659-023-00474-x)
Supplement: Supplementary file 1 — Additional file 1: Figure S1. TF targets analysis of DEGs in MX1-OE and EGR1 binding motif in AP-1 promoter region. [file 40659_2023_474_MOESM1_ESM.docx]

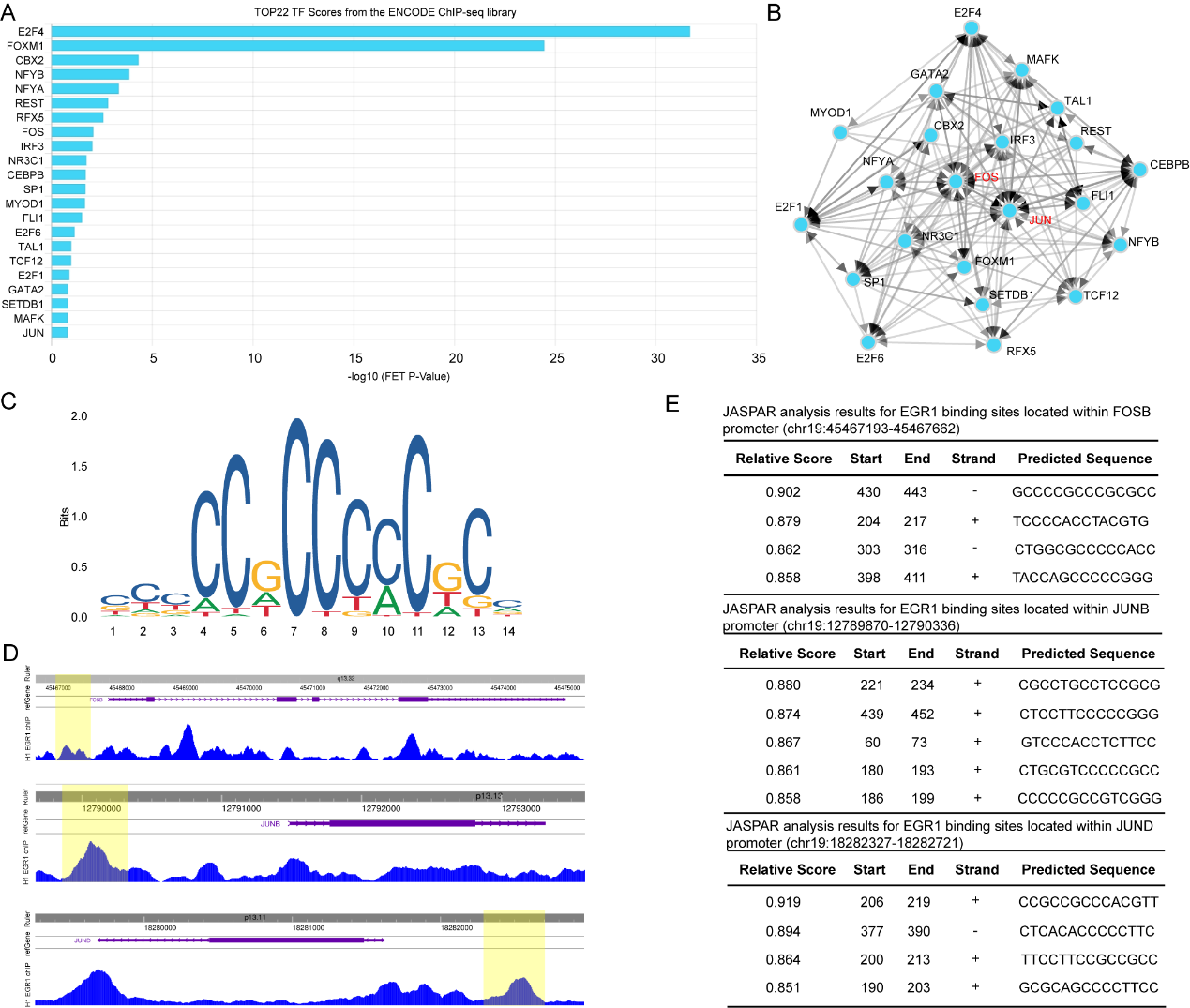


**Figure S1. TF targets analysis of DEGs in MX1-OE and EGR1 binding motif in AP-1 promoter region. (A)** The top 22 transcription factors targets for DEGs in MX1-OE from ENCODE ChIP-seq library. **(B)** The network of top 22 transcription factors targets for DEGs. **(C)** EGR1 motif from ENCODE ChIP-seq library (Matrix ID: MA0162.2). **(D)** EGR1 binding peaks in the promoter region of AP-1 TFs from CistromeDB (No:46169). **(E)** EGR1 binding motif analysis through JASPAR prediction.
